# NHX-Type Na^+^/H^+^ Antiporter Gene Expression Under Different Salt Levels and Allelic Diversity of *HvNHX* in Wild and Cultivated Barleys

**DOI:** 10.3389/fgene.2021.809988

**Published:** 2022-02-22

**Authors:** Zahra Jabeen, Faiza Irshad, Nazim Hussain, Yong Han, Guoping Zhang

**Affiliations:** ^1^ Department of Biosciences, COMSATS University Islamabad (CUI), Islamabad, Pakistan; ^2^ Department of Agronomy, Institute of Crop Science, College of Agriculture and Biotechnology, Zhejiang University, Hangzhou, China

**Keywords:** salinity stress, wild barley, gene expression, polymorphism, SNP, vacuole

## Abstract

Salinity tolerance is a multifaceted trait attributed to various mechanisms. Wild barley is highly specialized to grow under severe environmental conditions of Tibet and is well-known for its diverse germplasm with high tolerance to abiotic stresses. The present study focused on determining the profile of the expression of isoforms of the *HvNHX* gene in 36 wild and two cultivated barley under salt stress. Our findings revealed that in leaves and roots, expression of *HvNHX1* and *HvNHX3* in XZ16 and CM72 was upregulated at all times as compared with sensitive ones. The *HvNHX2* and *HvNHX4* isoforms were also induced by salt stress, although not to the same extent as *HvNHX1* and *HvNHX3.* Gene expression analysis revealed that *HvNHX1* and *HvNHX3* are the candidate genes that could have the function of regulators of ions by sequestration of Na^+^ in the vacuole. *HvNHX1* and *HvNHX3* showed a wide range of sequence variations in an amplicon, identified *via* single-nucleotide polymorphisms (SNPs). Evaluation of the sequencing data of 38 barley genotypes, including Tibetan wild and cultivated varieties, showed polymorphisms, including SNPs, and small insertion and deletion (INDEL) sites in the targeted genes *HvNHX1* and *HvNHX3*. Comprehensive analysis of the results revealed that Tibetan wild barley has distinctive alleles of *HvNHX1* and *HvNHX3* which confer tolerance to salinity. Furthermore, less sodium accumulation was observed in the root of XZ16 than the other genotypes as visualized by CoroNa-Green, a sodium-specific fluorophore. XZ16 is the tolerant genotype, showing least reduction of root and leaf dry weight under moderate (150 mM) and severe (300 mM) NaCl stress. Evaluation of genetic variation and identification of salt tolerance mechanism in wild barley could be promoting approaches to unravel the novel alleles involved in salinity tolerance.

## Introduction

Na^+^/H^+^, counter-transporters (NHXs) not only serve as essential membrane transporters but also help catalyze the neutral exchange of K^+^ or Na^+^ for H^+^ and have been found to play a significant role in pH and ion homeostasis, cell expansion, and salt tolerance. To develop crops with enhanced tolerance to abiotic stresses, the establishment of a better understanding of the underlying mechanisms is essential. With growing advances made within the last decade, researchers have revealed various mechanisms of adaptation and molecular details of responses triggered by salt stress. Although barley is tolerant to high salt stress compared to other cereals, its production is still hampered by salinity (Qui et al., 2011). Salinity is one of the most detrimental stress factors among the abiotic stresses (Stepien and Klobus, 2005) which affected nearly 800 and 32 million hectares of land around the world (Wani et al., 2020). Soil salinization causes severe reduction in barley production all around the world (Rengasamy et al., 2003; Mishra and Tanna 2017). In such a scenario, to go for soil amendments is near to impossible. To cope with soil salinity, the development of salt-tolerant cultivars could be the best possible solution. Hence, understanding salt-tolerant mechanisms is essential for the genetic improvement of crops. Having unique features among cereal crops, barley is widely used in physiological and genetic studies to unravel the mechanisms of salt tolerance (Munns and Mark, 2008). However, the process of domestication of the present cultivated barley (*H. vulgare* L.) resulted in loss of vital allelic variations (Russell et al., 2004), leading to limited genetic diversity in its gene pools in comparison to their wild ancestors. Therefore, a narrow genetic base could be a great obstacle in the development of cultivars more adapted to the environment (Ellis et al., 2000; Feuillet et al., 2008; Shavrukov et al., 2010). A wider range of genetic variations has been reported in barley populations under various stressful environments (Nevo et al., 1997). Moreover, Tibetan wild barley (*H. spontaneum* L.), which habited the Qinghai–Tibet Plateau of China years ago, is one of the ancestors of cultivated barley (Dai et al., 2012).

The involvement of both osmotic and ionic components has been elaborated in plant growth inhibition under salt stress (Munns et al., 2006), for example, Na^+^ specifies not only damage to leaf tissues but also causes many metabolic problems in plants due to its high accumulation in shoots (Munns et al., 2006). Low cytoplasmic Na^+^/K^+^ ratio is maintained by multiple mechanisms that include morphological and biochemical adaptations (Munns and Mark, 2008). Vacuolar Na^+^/H^+^ antiporters belonging to the NHX gene family, that is, *AtNHX1*, which serve to detoxify the cytoplasm by compartmentalization of Na^+^ into the vacuole, have emerged as a vital group of transporters that helped in Na^+^ tissue tolerance mechanisms (Pardo et al., 2006). According to previous studies, overexpression of the NHX gene and its homologs from other plant species could result in salt tolerance (Zhang and Blumwald 2001; He et al., 2005).

Based on subcellular localization and physiological roles, previous studies classified Na^+^ (K^+^)/H^+^ antiporters in plants into two families, namely, plasma membrane transporter (SOS) and intracellular transporter (NHX) (Brett et al., 2005). (Brett et al., 2005). The NHX family can be further divided into two distinct groups, class-I and class-II (Pardo et al., 2006). Class-I includes NHX isoforms of *A. thaliana* (*AtNHX1-4*) with strong vacuolar localization (Pardo et al., 2006; Barragan et al., 2012). However, NHX proteins belonging to class-II, namely, *AtNHX*5-6 exhibit endosomal localization in cells (Pardo et al., 2006; Rodríguez-Rosales et al., 2008). Cytosol is the main site of action for salts, affecting plant growth and development by disturbing important physiological and biochemical processes. Ion homeostasis, specific for each cellular compartment, is necessary for plant cells to ensure the availability of optimal conditions for gene expression, various enzymatic processes, and protein structure and function (Paroutis et al., 2004). Moreover, a range of physiological processes that include cell expansion, osmotic adjustment, ion regulation, pH homeostasis, and cellular stress responses are essential for plant cell homeostasis and are regulated by NHX-type (cation/H^+^) antiporters (Pardo et al., 2006; Bassil et al., 2012). These counter-transporters emerged in the early evolution of plants and have been found in entire sequences of plant genomes (Bassil et al., 2012; Chanroj et al., 2012).

The NHX family found in barley consists of four isoforms, mainly localized in the vacuole, called Na^+^/H^+^ antiporter genes (*HvNHX1* (AB089197.1), *HvNHX2* (AY247791), *HvNHX3* (DQ372061.1), and *HvNHX4* (DQ314285) (Francisco et al., 2012). Expression of genes responsive to salt stress could be species-dependent, and it starts from significantly lower salt levels, for example, 50–100 mM NaCl, which is sufficient for most of the plant species. However, depending on the degree of salt sensitivity, even lower concentrations could be considered for higher salt-sensitive plants. On the contrary, halophyte species could sustain their growth at higher salt levels (Shavrukov., 2013). Plants have developed various mechanisms to get rid of higher concentrations of Na^+^ ions, which include its transport from the cytosol into the vacuole or out of the cell with the help of Na^+^/H^+^ exchanger machinery found in the vacuolar and plasma membranes, respectively (Apse and Blumwald 2007). Thus, enhanced efficiency of the vacuole to compartmentalize more Na^+^ is a promising strategy to overcome both Na^+^ toxicity and osmotic effect caused by high salinity (Vera-Estrella et al., 2005).

However, this mechanism could not be the sole remedy of the problem as vacuoles have a limitation for accommodating Na^+^. Therefore, a combination of more mechanisms needs to work as a unit of the salt tolerance strategy. For instance, limiting Na^+^ entry and increasing Na^+^ extrusion could effectively reduce Na^+^ accumulation in the cytosol. As salt tolerance, barely, is a complex trait controlled by many factors (Zhu, 2001), only one trait is not likely to result in any significant improvement. The salinity problem can only be solved successfully if several important traits are combined in a complementary manner.

Tibet provides a rich gene pool of wild barley with wider variation in adaptation to abiotic stresses, including drought and salinity (Wu et al., 2011; Dai et al., 2012). For the discovery of novel alleles that are potentially related to salt tolerance in wild barley, evaluation of the genetic diversity and identification of salt tolerance mechanisms has been elaborated as important approaches (Wu et al., 2011). Moreover, among various available DNA molecular markers, SNPs are the most abundant type and have been proven to be useful in genetic studies. Barley seeds have been used in genotyping studies since long and also act as important resources for the characterization of genetic variation, which could ultimately pave the way for developing cultivated varieties with enhanced tolerance to abiotic stresses and other production challenges (Nevo and Chen, 2010). SNP markers were initially developed for cultivated barley; however, its usage could also be expanded to wild resources (Rostoks et al., 2005). In the present research, we aim to 1) highlight the salt tolerance mechanism of four isoforms, (*HvNHX1* to *HvNHX4*), located in the vacuole and differing in their level of K^+^ and Na^+^ accumulation and 2) determine allelic diversity of *HvNHX* isoforms in wild and cultivated barleys.

## Materials and Methods

This experiment was conducted in two parts. First, seeds of two cultivated barleys, CM72 (tolerant) Gairdner (sensitive), and two wild barleys, XZ16 (tolerant) and XZ169 (sensitive), were used to evaluate the salinity tolerance. Second, 36 wild barley accessions were used from eight sub-populations of Tibetan wild barley accessions to investigate the allelic variation on the basis of single-nucleotide polymorphism (SNP). The seeds were surface-sterilized with 3% H_2_O_2_ for 15 min and washed five times with double distilled water. The seeds were transferred to germination boxes and incubated (22/18°C, day/night) for 10 days. Uniform seedlings at the two-leaf stage were transplanted onto 35 L rectangular pots. Nutrient solution was prepared according to the method given by Zahra et al., (2014). The pH of the hydroponic solution was maintained at 5.5–6. After 2 weeks post transplantation, barley plants were grown in three different levels of salt stress, (1) 0 (control); (2) 150 mM NaCl; and (3) 300 mM NaCl. For gene expression analysis, root and leaf tissues were collected at four different time points between 0 and 24 h under 150 and 300 mM salt stress from four genotypes. A complete randomized design with three replications was used in this experiment.

### Determination of Sodium and Potassium Concentration in Plant Tissue

The plants were harvested after 3 weeks of salt treatment. Samples of roots and leaves were collected from plants of each treatment separately, and fresh weight was recorded. Similarly, dry weight was recorded by drying the plant samples at 70°C for 74 h respectively. For measurements of root and leaf sample Na^+^ and K^+^ concentrations, 0.1 g of dry root and leaf samples was dried to ash and then dissolved in 10 ml HNO_3_: H_2_O (1:1). An atomic absorption spectroscope (Shimadzu, Japan) was used for ion content measurements.

### RNA to cDNA Synthesis for RT- PCR

RNA from roots and leaves of four genotypes was extracted using a kit method (Tiangen Technology Co., Ltd.DP432), according to the manufacturer’s protocol. Full-length strand cDNA was synthesized from 2 μg of RNA using the Takara Bio Inc., (RR037A). BIO-RAD Master Mix kit was used to carry out RT-PCR reactions according to the manufacturer’s protocol. Measurements were taken for two biological and three technical repeats. The relative gene expression levels were calculated by subtracting the threshold cycle (Ct) values for *Gapdh* from those of the target gene (to give ΔCt) and then calculating 2^−△△Ct^ (Livak and Schmittgen, 2001). The primers used for RT-PCR were designed using Primer-BLAST (Supplementary Table S1).

### Isolation and Sequence Analysis of *HvNHX1* and *HvNHX3* cDNA

After confirmation of the gene expression analysis, out of four isoforms, two (*HvNHX1* and *HvNHX3*) were selected to further investigate allelic variation based on SNPs and small INDEL detection approach. To detect SNPs in the cDNA pool from 38 barley genotypes, four primer pairs on to *HvNHX1* (Supplementary Figure S1; Supplementary Table S2) and three pairs of primers to the *HvNHX3* gene were designed to amplify whole coding sequence (CDS) regions for sequencing (Supplementary Figure S2; Supplementary Table S2). PCR primers were designed using Primer-BLAST. Each 50 μL PCR was carried out using 25 μl (2x Easy Taq^®^ PCR Super Mix), 2 μl (cDNA template), 1/1 μl (Primer forward/reverse 10 μM), and 21 μl (ddH_2_O). To confirm the primer amplification specificity, samples were tested for gel electrophoresis, and the required bandwidth obtained was then matched with a wide range of DNA markers. The samples were then sent to Shanghai Majorbio Co. Ltd., Shanghai, P.R. China for sequencing. Gene-specific primers were used for SNP identification. The sequences obtained from the company were aligned using ClustalX software to observe SNPs.

### Visualization of Na^+^ Ions Through Fluorescence Dye

To observe tissue-specific Na ion accumulation, the roots were stained with 25 mM specific fluorescent probe (Coro Na-Green AM) and 0.02% pluronic acid (Invitrogen) for 3 h as described by in our previous study by Zahra et al., (2014). The roots were incubated in the dark for 3 h and then root tips were carefully washed with deionized water and observed using a confocal microscope (LSM 710 NLO Jena, Germany) at wavelengths of 492 and 516 nm.

### Statistical Analysis

SPSS (17.0) software was used for data analyses. The sequences were assembled using DNAStar. TASSEL was used to identify single-nucleotide polymorphisms (SNPs) within the sequence of the *HvNXH1* and *HvNXH3* genes.

## Results

### The Difference in Tissue Dry Weight and Ion Content Among Four Genotypes Under Moderate and Severe Salt Stress

After 3 weeks of salt treatment, the treated plants showed a significant reduction in root and leaf dry weight; however, the level of reduction varied among the four genotypes (Table 1). XZ16 and CM72 are more tolerant genotypes, showing a minimal reduction of root and leaf dry weight under moderate (150 mM) and severe (300 mM) NaCl stress, whereas the two salt-sensitive genotypes, Gairdner and XZ169, exhibited a significanty reduction in root and leaf dry weight reduced relative to the control. However, the extent of reduction in dry weight of roots and leaves at both salinity levels was in order of XZ16 < CM72 < XZ169 < Gairdner (Table 1).

**TABLE 1:** Comparison of root and leaf dry weight, Na^+^ and K^+^ content, and the Na^+^/K^+^ ratios among four barley genotypes under salt stress.

Salinity Levels (mM)	Genotypes	DW (g) roots and leaves		K^+^(mg g^−1^ DW) roots and leaves		Na^+^ (mg g^−1^ DW) roots and leaves		Na^+^/K^+^ ratio roots and leaves	
**0**	**XZ16**	0.19	1.26	62.16	66.70	4.140	5.303	0.06	0.08
	**CM72**	0.18	1.25	64.86	66.94	2.850	3.29	0.04	0.05
	**XZ169**	0.18	1.23	64.99	68.90	3.54	4.83	0.05	0.07
	**Gairdner**	0.17	1.25	70.73	71.07	3.38	4.00	0.05	0.06
**150**	**XZ16**	0.17(11%)	1.13(10%)	18.65	45.73	20.53	35.95	1.10	0.8
	**CM72**	0.16(11%)	1.12 (10%)	17.93	39.05	23.78	36.57	1.33	0.9
	**XZ169**	0.13(28%)	0.77(38%)	14.74	24.12	35.32	65.83	2.39	2.7
	**Gairdner**	0.12(29%)	0.66(47%)	12.96	21.98	49.38	68.58	3.82	3.1
**300**	**XZ16**	0.13(24%)	0.71(37%)	9.43	30.75	35.75	52.42	3.79	1.7
	**CM72**	0.12(25%)	0.68(39%)	9.04	29.88	43.87	62.20	4.85	2.1
	**XZ169**	0.09(31%)	0.36(53%)	6.29	16.21	55.55	91.38	8.82	5.5
	**Gairdner**	0.06(50%)	0.32 (51%)	5.95	14.27	62.53	96.48	10.50	6.7
	***p*** **level**	**	**	**	**	**	**	**	**

Probability level (*p*)^**^ significant at *p* ≤ 0.01. Bold values indicate the genes position.

Na^+^ concentrations in both roots and leaves of four genotypes were noticeably enhanced under salt stress, while K^+^ concentrations in roots and leaves of the four genotypes showed a remarkable reduction with increasing NaCl levels. Hence, more increase of Na^+^ concentration and reduction of K^+^ concentration was observed in Gairdner and XZ169 than that in XZ16 and CM72. Therefore, Gairdner and XZ169 had a considerably higher Na^+^/K^+^ ratios than XZ16 and CM72 in both roots and leaves under moderate and severe salt stress (Table 1).

### Correlation Between Relative dry Weight and Ionic Contents

**FIGURE 1:**
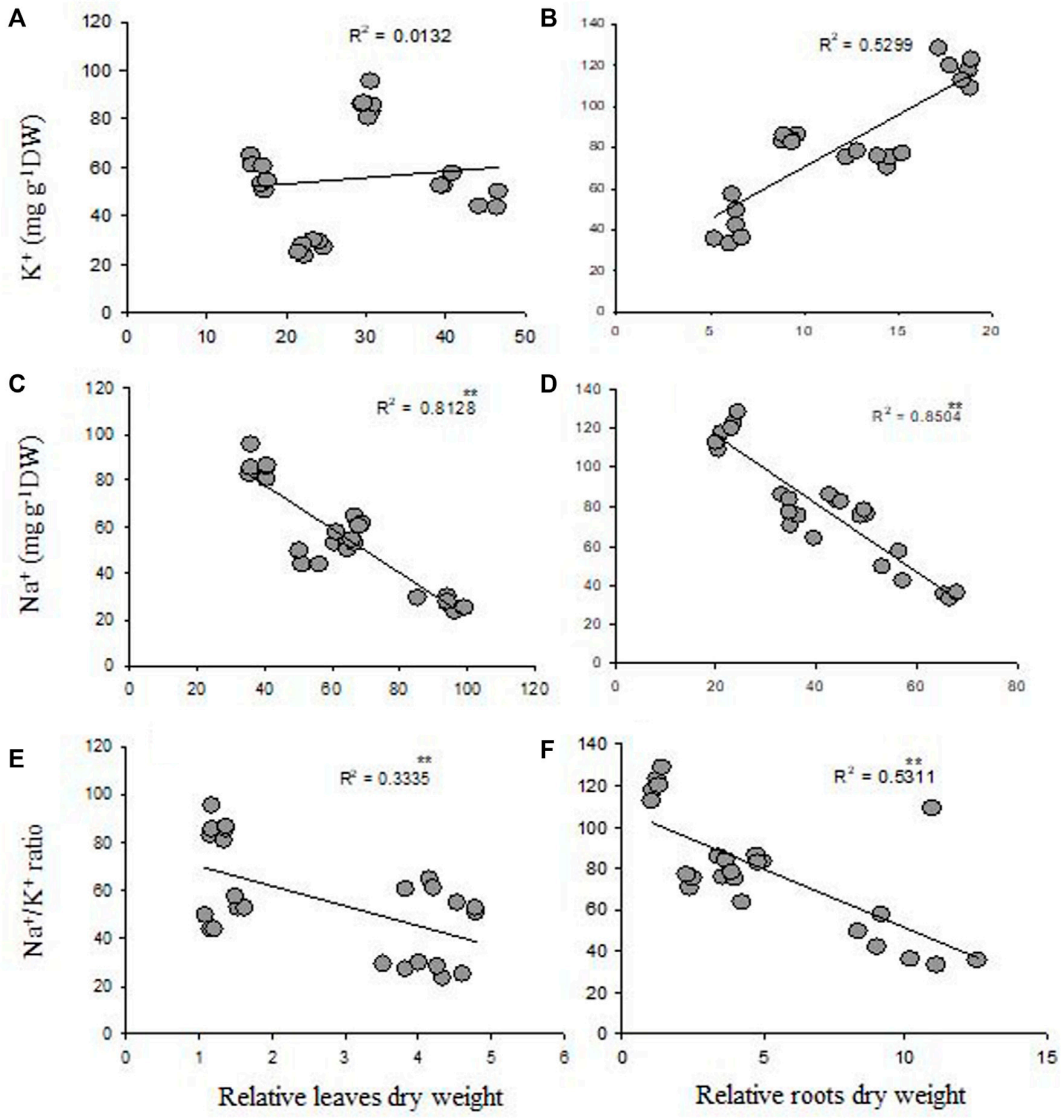
Correlation between K^+^ and Na^+^ contents and Na^+^/K^+^ ratios and relative leaf **(A,C,E)** and root **(B,D,F)** dry weight is based in of four genotypes.

Na^+^ concentrations in leaves and roots were significantly negatively correlated with relative root and leaf dry weight (Figures 1C,D). Moreover, K^+^ concentration in both the tissues had a positive correlation with relative leaf and root dry weight (Figures 1A,B). However, a significantly negative correlation was observed between shoot and root Na^+^/K^+^ ratio and relative leaf and root dry weight (Figures 1E,F).

### Expression of Na^+^/H^+^-Antiporters (*HvNHX*) Isoform in Response to Moderate and Severe Salt Stress in Roots

**FIGURE 2:**
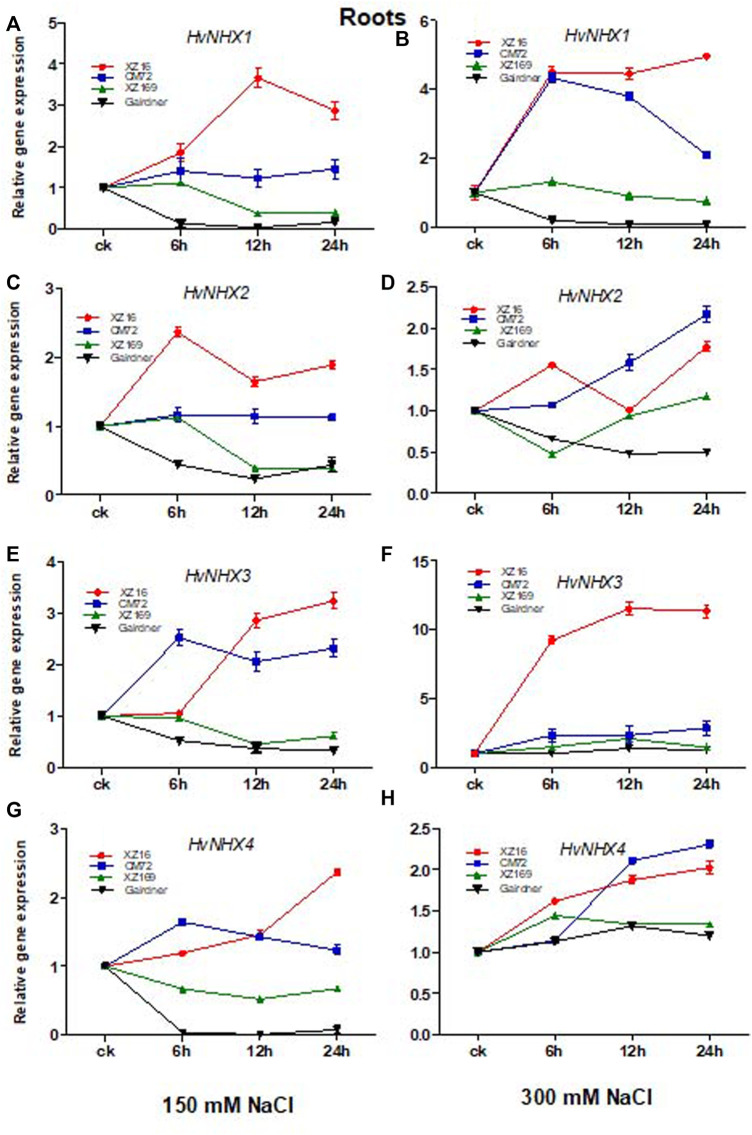
Relative gene expression of *HvNHX* isoforms in leaves of four genotypes after moderate and severe salt stress. Data are expressed asmeans ± SD of at least three repeats.

We studied the influence of salt stress on *HvNHX1*, *HvNHX2*, *HvNHX3*, and *HvNHX4* gene expression in roots and leaves of four barley genotypes after 0, 6, 12, and 24 h of exposure to moderate and severe salt stress. Our results revealed that in the case of XZ16, the *HvNHX1* gene showed a higher expression level in the root tissue at all time points under moderate and severe salt stress than the control. The expression level of the *HvNHX1* gene in CM72 was also upregulated at all time points under moderate and severe salt stress; however, the expression level of *HvNHX1* in XZ16 was markedly enhanced as compared with CM72 (Figures 2A,B), whereas in salt-sensitive genotypes XZ169 and Gairdner, downregulation was observed in the expression level of the *HvNHX1* gene at all time points in XZ169, except at 6 h, whereas in Gairdner at both the salinity levels, downregulation was observed as compared with the control (Figures 2A,B).

The expression level of *HvNHX2* was significantly upregulated in XZ16 at both salinity levels as compared with the control, but a higher expression level was observed at 6 and 24 h (Figures 2C,D) However, the expression level of the *HvNHX2* gene in CM72 under moderate salt stress was slightly expressed as compared with the control, but at 12 and 24 h exposure of severe stress, the expression level in CM72 was more than that of XZ16 under 300 mM salt stress. In contrast, the expression level of *HvNHX2* in Gairdner was downregulated under both salinity levels, while the expression level in XZ169 was not affected at the initial hour but then declined under moderate salt stress, while the reverse was true for severe salt stress (Figures 2C,D).

It was also found that the expression of the *HvNHX3* gene in XZ16 was highly upregulated at both salinity levels as compared with other three genotypes, whereas the expression level in CM72 was upregulated in moderate salt stress, while at severe salt stress, slight increase was observed as compared with the control (Figures 2E,F). Moreover, the expression level in XZ169 and Gairdner was downregulated at moderate salt stress, and no change was observed in *HvNHX3* gene expression in response to 300 mM NaCl (Figures 2E,F).

The expression level of *HvNHX4* was upregulated in XZ16 and CM72 under both levels of salt stress, but the expression level was higher in XZ16 than CM72 under moderate salt stress, whereas the reverse was true for CM72 under severe salt stress. In the roots of XZ169 and Gairdner, the expression of *HvNHX4* was downregulated at moderate salt stress, whereas it was slightly upregulated under severe salt stress (Figures 2G,H).

### Expression of Na^+^/H^+^-Antiporter (*HvNHX*) Isoform in Response to Moderate and Severe Salt Stress in Leaves

**FIGURE 3:**
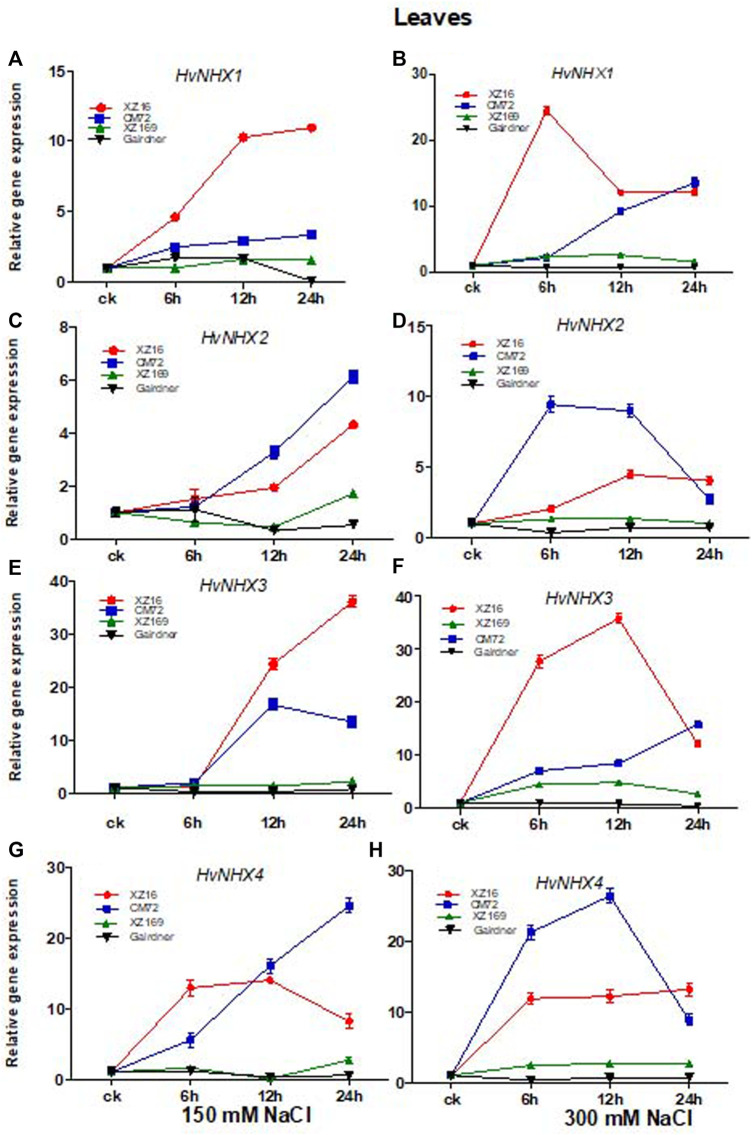
Relative gene expression of *HvNHX* isoforms in roots of four genotypes after moderate and severe salt stress. Data are expressed as means ± SD of at least three repeats.

The expression level of *HvNHX1* in leaves of XZ16 and CM72 was upregulated at all time points, but markedly increased in XZ16 compared with CM72 under moderate and severe salt stress (Figures 3A,B). In contrast, the expression level of *HvNHX1* in Gairdner was downregulated at 24 h of moderate salt stress, while no change was observed at severe salt stress compared with the control. However, the expression level in XZ169 was slightly upregulated under both salinity levels (Figures 3A,B Figures 3A,B). Moreover, the expression level of *HvNHX2* was significantly upregulated in XZ16 and CM72 at both salinity levels. However, this gene was more highly expressed in CM72 than XZ16 under both salinity levels, except at 24 h of severe salt stress (Figures 3C,D). In contrast, *HvNHX2* in Gairdner was downregulated under both salinity levels, whereas the expression level in XZ169 was slightly increased at 24 h of moderate salt stress as compared with the control (Figures 3C,D). As shown in Figures 3E,F, the expression of the *HvNHX3* gene in XZ16 was highly upregulated at both salinity levels as compared with the other three genotypes, except at 24 h of severe salt stress, where expression of *HvNHX3* was slightly downregulated as compared with CM72. In contrast, the expression of *HvNHX3* in Gairdner was downregulated under both salinity levels. Moreover, the expression level in XZ169 remained unchanged under moderate salt stress but slightly induced at 6 and 12 h and then declined at 24 h of severe salt stress as compared with the control (Figures 3E,F).

The expression of *HvNHX4* was upregulated in XZ16 and CM72 under moderate and severe salt stress as compared with the control. The expression was more pronounced in CM72 than XZ16, except at 24 h of severe salt stress in ZX16. In leaves, the overall expression level of *HvNHX4* in XZ169 remained unchanged but slightly induced at 6 h under moderate salt stress, whereas under severe salt stress, the expression of *HvNHX4* in XZ169 was slightly upregulated at all time points (Figures 3G,H).

In general, both moderate and severe salt treatment caused fluctuations in the expression of all *HvNHX* isoforms in roots and leaves of both the salt-tolerant genotypes as compared with sensitive ones. The strongest expression level in XZ16 roots and leaves was ranked as *HvNHX1>HvNHX3>HvNHX4>HvNHX2* and in CM72 as *HvNHX2*>*HvNHX4*> *HvNHX1*>*HvNHX3*. *HvNHX* isoforms were also induced in XZ169, the salt-sensitive genotype, but to a lesser extent. However, all the *HvNHX* isoforms were down regulated in Gairdner.

**FIGURE 4:**
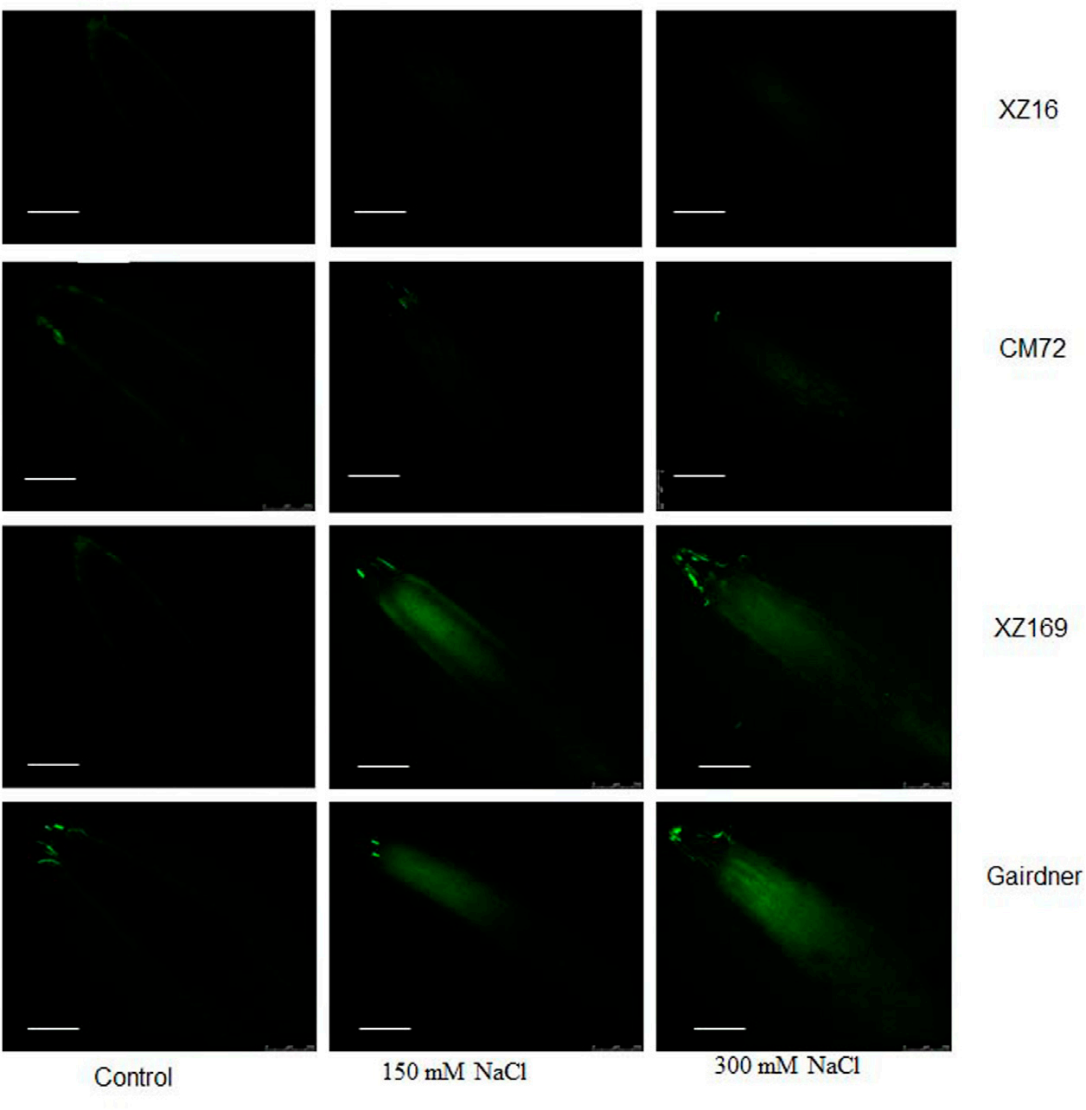
Fluorescence detection of Na^+^ accumulation in roots of four genotypes after moderate and severe salt stress. Scale bar10 µm.

It was also observed that less Na^+^ was accumulated in the roots of XZ16 than the other genotypes visualized by CoroNa-Green, a sodium-specific fluorophore (Figure 4). Bright images showed that more Na^+^ content was accumulated in plant roots.

### SNP Detection and Validation

**FIGURE 5:**
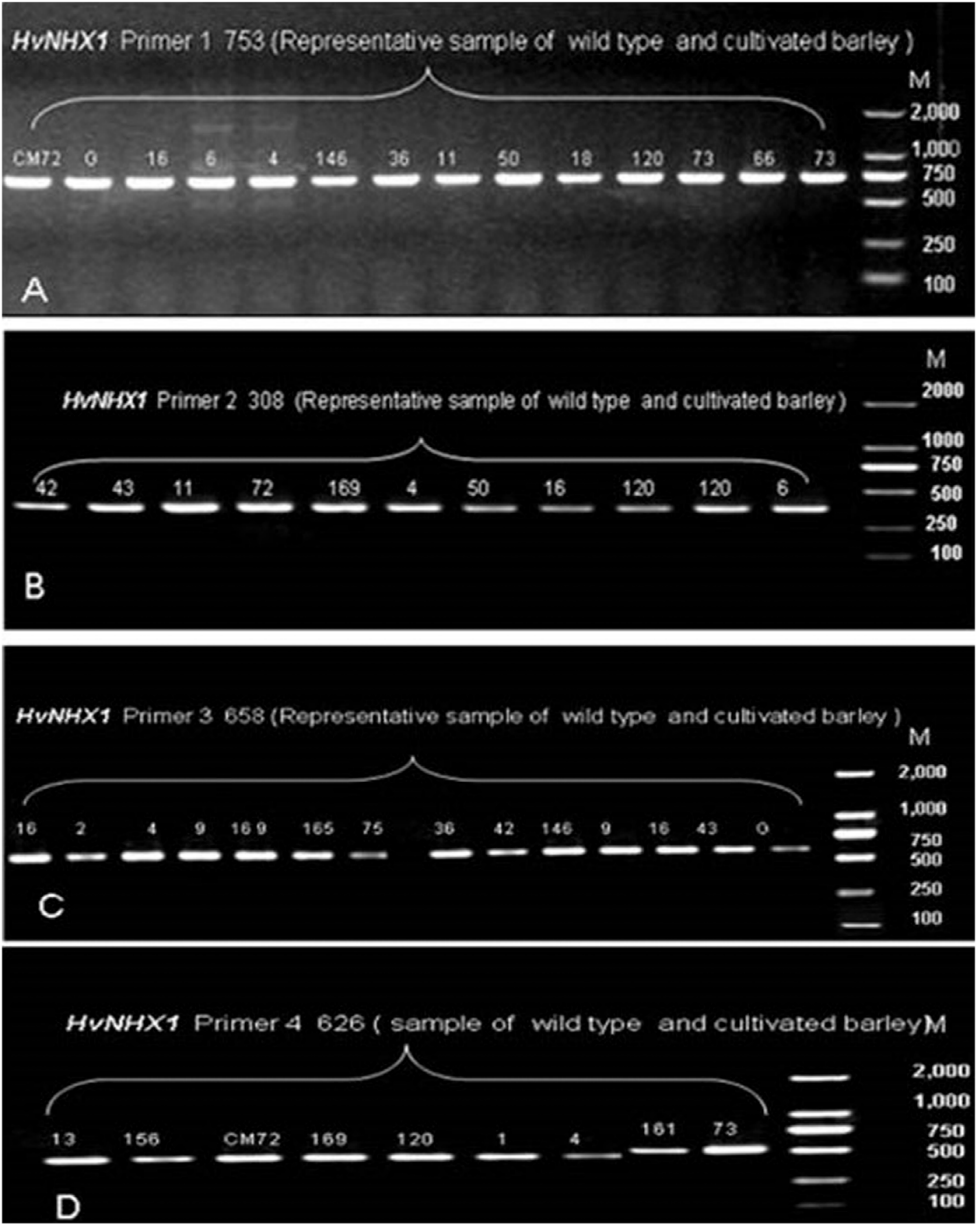
Pre-sequencing sample amplification trial to verify the gene and amplicon size for the selected primer pairs of *HvNHX1* primer 1 **(A)**, primer 2 **(B)**, primer 3**(C)**, and primer 4**(D)**. M = wide range DNA marker (100–2000 bp).

**FIGURE 6:**
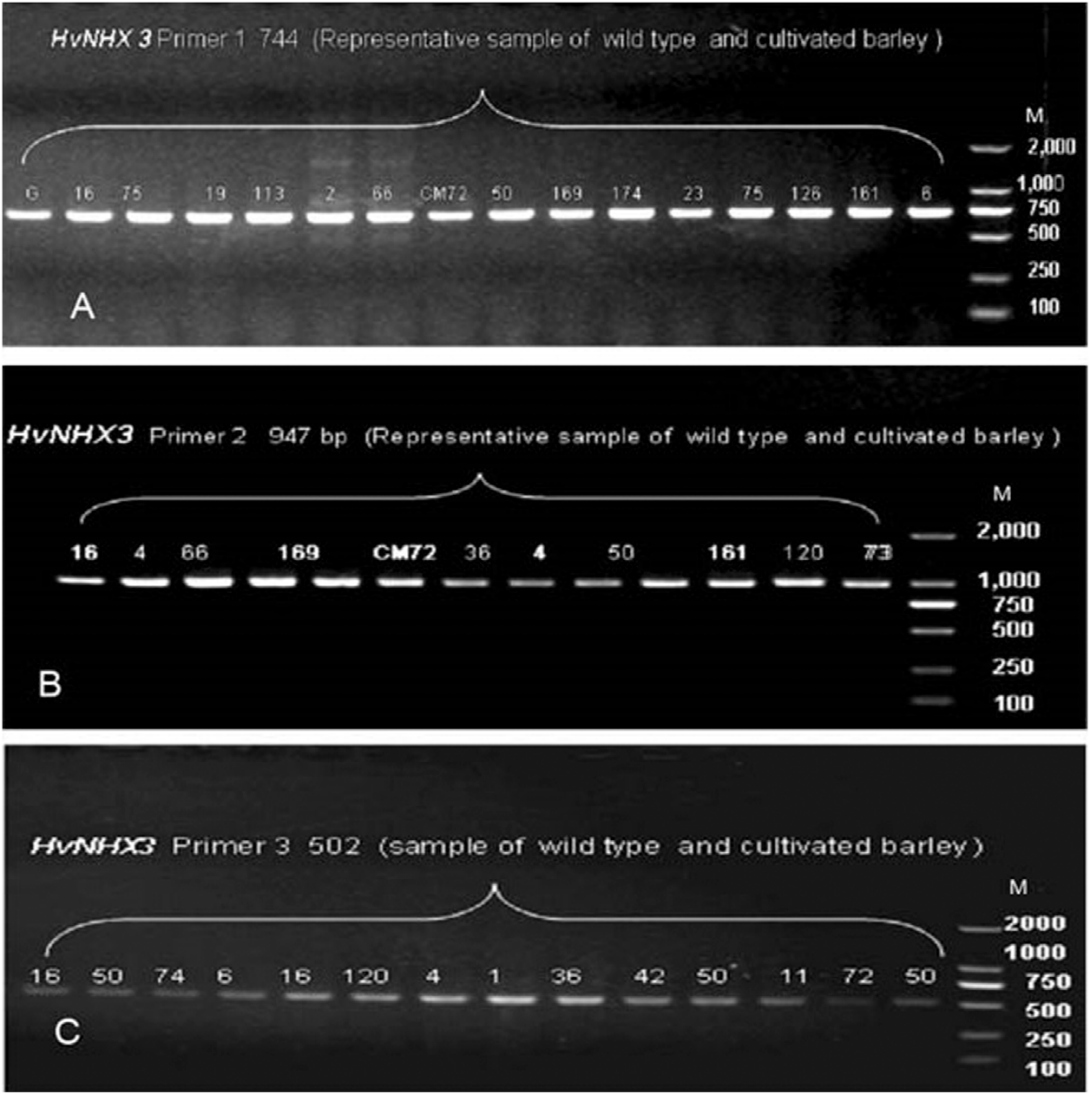
Pre-sequencing sample amplification trial to verify the gene and amplicon size for the selected primer pairs of *HvNHX3* primer 1 **(A)**, primer 2 **(B)**, and primer 3**(C).** M = wide range DNA marker (100–2000 bp).

In the present study, 36 wild barley accessions (from eight sub-population of Tibetan wild barley accession) and two cultivated barley genotypes were used to investigate the allelic variation in *HvNHX1* and *HvNHX3* based on SNPs. The sequence of genes *HvNHX1* (AB089197.1) and *HvNHX3* (DQ372061.1) was downloaded from NCBI. Complete coding regions of mRNA of *HvNHX1* (2564 bp) and *HvNHX3* (1794 bp) were cloned. Four primer pairs to *HvNHX1* and three pairs of primers to the *HvNHX3* gene were designed to amplify the complete CDS regions of candidate genes for sequencing. In order to confirm the primer amplification specificity, few of the representative samples were tested for gel electrophoresis, and the required bandwidth obtained was then matched with a wide range of DNA markers (Figures 5, 6). SNPs were detected using the sequencing and alignment method. We successfully amplified and sequenced *HvNHX1* and *HvNHX3* genes. Details of the nucleotide variations among the barley accessions and/or genotypes for *HvNHX1* genes are presented in Table 2 and *HvNHX3* genes are presented in Table 3.

**TABLE 2:** Sequencing results showing single-nucleotide polymorphisms (SNPs) and insertion–deletions (INDEL) pattern in the *HvNHX1* gene locus having Na^+^/H^+^ activity in barley; del: deletion. Dots indicate the same nucleotide with the reference sequence; the letters in each sample represent nucleotide substitution sites. SPS = SNP per sample. The letters in each sample represent nucleotide substitution sites.

Gene position (5′ to 3′)																		
	267	300	340	695	825	1069	1816	1818	1820	1827	1588	1741	1828	1843	1936	1978	2010	SPS
REF-HvNHX1	G	A	A	C	A	A	C	C	G	A	G	G	G	C	T	A	C	
CM72	.	del	.	.	.	.	.	.	.	.	.	.	.	.	.	G	.	1
G	del	.	.	.	.	del	del	.	.	.	.	.	.	.	.	.	.	0
XZ1	.	.	.	.	.	.	.	.	.	.	.	.	.	T	.	.	.	1
XZ2	.	.	.	.	C	.	.	.	.	.	.	.	.	.	.	.	G	2
XZ4	A	.	.	.	.	.	.	.	.	.	.	.	.	.	.	.	.	1
XZ6	.	.	.	.	.	.	.	.	.	.	.	.	.	.	.	.	.	0
XZ9	.	.	.	.	.	.	del	.	A	.	A	.	.	.	.	.	G	3
XZ11	.	.	.	.	.	.	.	del	.	.	.	.	.	.	.	.	.	0
XZ13	del	.	.	.	C	.	.	del	.	.	.	.	.	.	.	.	.	1
XZ16	.	.	.	.	.	.	.	.	.	.	.	.	.	T	.	.	.	1
XZ18	.	.	T	.	.	.	.	.	.	.	.	A	.	T	G	.	.	3
XZ23	A	.	.	T	.	.	del	del	.	G	.	.	A	.	.	.	.	4
XZ36	del	.	.	.	.	G	.	.	.	.	.	.	A	.	.	.	.	2
XZ38	del	.	.	.	.	.	.	.	.	.	.	.	.	.	.	.	.	0
XZ41	.	.	.	.	.	.	.	.	.	.	.	.	.	.	.	.	.	0
XZ42	.	del	.	.	.	G	.	.	.	.	.	.	.	.	.	.	.	1
XZ50	del	del	.	.	.	.	.	.	.	.	.	.	.	.	.	.	G	1
XZ61	.	.	.	.	.	.	.	.	.	.	.	.	.	.	.	.	.	1
XZ66	del	.	.	.	.	.	.	.	.	.	.	.	.	T	.	.	del	1
XZ73	.	.	.	.	.	.	del	del	.	G	.	.	.	.	.	.	.	1
XZ74	.	.	.	.	.	.	.	.	.	.	.	.	.	.	.	.	.	0
XZ75	.	.	.	.	.	G	.	.	.	.	.	.	.	.	.	.	.	1
XZ78	.	del	.	.	.	del	.	.	.	.	.	.	.	.	.	.	G	1
XZ87	.	.	.	.	.	.	.	.	.	.	.	.	.	.	.	.	G	1
XZ113	.	.	.	.	.	.	.	.	.	.	.	.	A	.	.	.	.	1
XZ115	.	.	.	.	C	.	.	.	.	.	.	.	.	.	.	.	.	1
XZ120	.	.	.	.	.	.	.	.	.	.	.	.	.	T	.	.	G	2
XZ126	T	del	.	.	.	.	.	.	.	G	.	.	.	.	.	.	.	2
XZ146	.	.	.	.	.	G	.	.	.	.	.	.	.	.	.	.	.	1
XZ156	.	.	.	.	.	.	del	.	.	.	.	.	.	.	.	.	.	0
XZ161	.	.	.	.	.	.	.	.	.	.	.	.	.	.	.	.	del	0
XZ165	del	.	.	.	.	.	.	del	.	.	.	.	A	.	.	.	.	1
XZ166	.	.	.	.	.	.	.	.	.	.	.	.	.	.	.	.	.	0
XZ167	.	.	.	.	.	.	.	.	.	.	.	.	A	.	.	.	.	1
XZ169	.	.	.	.	.	.	del	.	A	.	.	.	.	.	.	.	.	1
XZ174	.	.	.	.	.	.	.	.	.	.	.	.	A	.	.	.	.	1
XZ179	.	.	.	.	.	.	del	del	.	G	.	.	.	.	.	.	.	1
XZ188	.	.	.	.	.	.	.	del	.	.	.	.	.	.	.	.	.	0

**TABLE 3:** Sequencing results showing single-nucleotide polymorphisms (SNPs) and insertion–deletion (INDEL) pattern in the HvNHX3 gene locus having Na^+^/H^+^ activity in barley. H: haplotype; del: deletion. Dots indicate the same nucleotide with the reference sequence; the letters in each sample represent nucleotide substitution sites. SPS = SNP per sample. The letters in each sample represent nucleotide substitution sites.

Gene position (5′ to 3′)													
	11	45	373	736	992	1,121	1,147	1,150	1,190	1,221	1,455	1,661	SPS
REF-HvNHX3	G	G	A	C	G	C	A	G	T	T	C	G	
CM72	.	.	T	.	.	.	.	.	.	G	.	.	2
G	.	.	T	.	.	.	.	.	.	.	.	.	1
XZ1	T	.	T	T	.	.	.	.	del	del	.	.	3
XZ2	.	.	T	T	.	.	.	.	.	.	.	.	2
XZ4	.	.	T	T	.	.	.	.	del	.	.	.	2
XZ6	T	.	T	T	.	.	.	.	A	.	.	.	4
XZ9	T	.	T	.	.	.	.	.	.	.	.	.	2
XZ11	.	C	T	del	.	.	.	.	.	G	.	A	4
XZ13	T	C	T	.	.	.	.	.	.	.	.	.	3
XZ16	del	T	T	.	.	.	.	.	.	G	.	A	4
XZ18	del	del	T	del	.	.	.	.	.	.	.	.	1
XZ23	.	.	T	C	.	.	.	.	del	.	.	.	2
XZ36	.	.	T	T	.	.	.	.	.	.	.	A	3
XZ38	.	.	.	.	.	.	.	.	.	.	.	.	0
XZ41	.	.	T	T	.	.	.	.	del	.	.	A	3
XZ42	.	.	T	del	.	.	.	.	.	.	.	.	1
XZ50	.	.	T	.	.	.	.	.	.	.	.	.	1
XZ61	del	.	T	.	.	.	.	.	del	.	.	.	1
XZ66	T	.	T	del	.	.	.	.	del	.	.	.	2
XZ73	.	.	T	del	.	.	.	.	.	G	.	A	3
XZ74	.	.	T	del	.	.	.	.	.	.	.	.	1
XZ75	.	.	T	T	.	.	.	.	.	.	.	.	2
XZ78	.	.	T	.	C	A	C	T	del	.	.	.	5
XZ86	.	.	T	del	.	.	C	.	.	.	.	.	2
XZ113	.	.	T	del	C	.	.	.	del	.	.	.	2
XZ115	.	.	T	T	.	.	.	T	.	.	.	.	3
XZ120	.	.	T	.	C	A	.	.	.	.	.	.	3
XZ126	.	.	T	T	.	.	.	.	.	.	.	.	2
XZ146	.	.	.	.	.	.	.	.	.	.	.	.	0
XZ156	.	.	T	T	.	.	.		.	del	.	.	2
XZ161	del	.	T	.	.	A	C	T	.	.	.	.	4
XZ165	.	.	T	T	.	.	.	.	del	.	.	.	2
XZ166	.	.	T	T	.	.	.	.	.	A	.	.	3
XZ167	.	del	T	del	.	.	.	.	A	del	T	.	3
XZ169	T	.	T	.	.	.	.	.	del	.	.	.	2
XZ174	T	.	T	.	.	.	.	.	.	.	.	.	2
XZ179	.	del	T	del	.	.	.	.	.	.	.	.	1
XZ188	.	.	T	del	.	.	.	.	del	.	.	.	1

Overall, evaluation of the sequencing data of 38 barley genotypes showed mutation, including SNPs and small insertion–deletion (INDEL) sites, for targeted gene *HvNHX1.* Thirty-nine SNPs were observed in total 38 barley genotypes/accessions as shown in Table 2. Moreover, the range of polymorphic sites was 1–3, with an average of one SNP site per barley genotype (Table 2). The *HvNHX3* gene was amplified by three primers. The range of polymorphic sites was 1–5, with an average of two SNP sites per barley genotype. Eighty-four SNPs were detected for the *HvNHX3* gene in 38 barley genotypes/accessions as shown in Table 3.

Moreover, SNP analysis revealed that in CM72 (salt-tolerant genotype), A is deleted at 300bp and replaced by G at 1978bp while in XZ16 (salt-tolerant genotype), C was replaced by T at 1843bp. For salt-sensitive Gairdner genotype, G, A, and C were deleted at 267, 1,069, and 1,816 bp, respectively. In wild XZ169 salt-sensitive genotype, C was deleted at 1,816 bp and G was replaced by A at 1,820 bp as compared with the reference *HvNHX1* gene. Moreover, in the *HvNHX3* gene in CM72, A and T was replaced by T and G at 373 and 1221bp, respectively, while in XZ16, G was deleted at 11bp and replaced by T at 45bp while A, T, and G were replaced by T, G, and A at 373, 1,221, and 1,661 bp, respectively (Table 3). For salt-sensitive Gairdner genotype, A was replaced by T at 373bp. In XZ169, G was replaced by T at 11 bp while A was replaced by T at 373 bp and at 1190 bp. T was deleted as compared with the reference *HvNHX3* gene. It may be concluded that variations of SNP in salt-tolerant wild barley might offer elite alleles for the development of salt-tolerant barley.

## Discussion

Salinity tolerance in plants is a complex multigenic trait, including physiological and molecular aspects (Flowers, 2004). In salt stress conditions, Na^+^ first enters the cytosol of a plant and disturbs important physiological, biochemical, and molecular processes; consequently, it restricts plant growth, disruption of ion homeostasis (Mane et al., 2010; Basu et al., 2020), and development, therefore posing a serious threat to crop production (Zhu, 2007). One of the key mechanisms in plants to cope with salinity stress is their ability to reduce sodium ion (Na^+^) transport at both the tissue and cellular level, either by emitting Na^+^ into tissues (Tester and Davenport 2003), or by maintaining ion homeostasis within the cell, including Na^+^ compartmentalization in the vacuole (Tester and Davenport 2003; Flowers and Colmer 2008). Barley is a well-known salt-tolerant crop (Steppuhn et al., 2005; Jabeen et al., 2021); however, in cultivated barley, the increase in sensitivity to salinity stress is observed due to the increasingly narrow genetic diversity (Zhu, 2001). However, the Tibet plateau provides a rich pool of wild barleys with a high degree of contrast in salt tolerance that could be attributed to high genetic diversity compared with cultivated barley (Shavrukov et al., 2010).

In this study, the salt tolerance mechanism of four isoforms (*HvNHX1* to *HvNHX4*) and their allelic diversity in wild and cultivated barleys was evaluated after application of 150 and 300 mM salt stress in hydroponic conditions. Salt stress causes several physiological, morphological, and biochemical changes in plants (Uçarlı and Gürel 2020). The current results indicate that XZ16 and CM72 are more tolerant genotypes with minimal reduction of root and leaf dry weight under moderate (150 mM) and severe (300 mM) NaCl stress. However, within tolerant genotypes, wild barley XZ16 showed comparatively less reduction than cultivated barley CM72. This suggested the involvement of a rather different mechanism of salt tolerance in XZ16 compared with CM72. The result was consistent with the findings observed in our previous study (Wu et al., 2011; Zahra et al., 2014). A high expression level of Na^+^/H^+^ antiporters in tolerant plants could lead to enhanced Na^+^ compartmentalization into vacuoles and ultimately improve plant growth by defending the cytoplasm from harmful effects of Na^+^ (Blumwald et al., 2000; Galvez et al., 2012). Moreover, in the present study, the increase in Na^+^ and decrease in K^+^ concentrations in Gairdner and XZ169 was distinctly higher than those in XZ16 and CM72. A similar trend was observed in the studies conducted by Qui et al., 2011; Wu et al., 2011, describing that the reduction in growth was caused by enhanced Na^+^ and reduced K^+^ tissue content, which caused ion toxicity and damaged plant metabolism and growth (Table 1).The tissue-specific Na ion accumulation was also confirmed through fluorescence dye, which was directly proportional to the Na^+^ ion accumulation in roots (Figure 4). Genotypes with the lowest Na^+^ tissue accumulation produced more biomass and vice versa (Munns and Mark, 2008). In the present study, the expression level of *HvNHX* isoforms follows a complex pattern, but the gene expression was more induced in salt tolerant genotypes, indicating the important role of these genes in the salt tolerance mechanism. In leaves and roots, the expression of *HvNHX1* and *HvNHX4* in XZ16 and CM72 was upregulated at all time points as compared with sensitive ones, NHX1 (Quintero et al., 2009; Fukuda et al., 2004; Saqib et al., 2005; Brini et al., 2007) and *NHX4* (Gálvez et al., 2012) in wheat and Arabidopsis, and a high expression pattern of *NHX1* (Quintero et al., 2009; Fukuda et al., 2004; Saqib et al., 2005; Brini et al., 2007) and *NHX4* genes in tomato (Galvez et al., 2012) was reported to be involved it in better plant growth because *NHX1* and *NHX4* are the key molecular players in maintaining plant cell homeostasis by regulating several physiological processes, such as cell expansion, osmotic adjustment, cell volume, pH, and ion regulation (Pardo et al., 2006).

Na^+^/H^+^ antiporter protein has different membrane positions in the cell, and its function may be affected by ion accumulation. Previously, the topological studies elucidated that the position of N-terminal of *AtNHX1* is facing toward the cytosol, and its C-terminal, hydrophilic region, residing in the vacuolar lumen could protect the cytoplasm from deleterious effects of Na^+^ (Yamaguchi and Blumwald, 2005, Flowers and Colmer, 2008. The function of Na^+^/H^+^ antiporters may not only be related to the regulation of gene expression but could also be involved in transcriptional modification of the proteins. The activity of antiporters could be regulated by phosphorylation through the interaction of various kinases with other cellular proteins. So, the differential response of these binding factors in a species-dependent manner could alter the activity of the Na^+^/H^+^ antiporters. For instance, the binding of *AtCaM15*, a calmodulin-like protein 15 (localized in plant vacuolar compartment), to the C-terminal domain of *AtNHX1* (a tonoplast transporter) changed the Na^+^/K^+^ selectivity of the antiporter in Arabidopsis (Yamaguchi and Blumwald, 2005; Munns and Mark, 2008). Moreover, similar to the overexpression of *HvNHX1* in tolerant genotypes, *HvNHX3* was also expressed in wild barley XZ16 and CM72. These results suggest that *HvNHX1* and *HvNHX3* may encode a putative vacuolar *NHX* that could play an important role in salt tolerance by mediating K^+^/H^+^ exchange in plants (Liu et al., 2010).

In plants, H^+^V-PPase and H^+^-ATPase are the two different vacuolar pumps that help the Na^+^/H^+^ antiporter in the vacuolar lumen to transport Na^+^ from the cytoplasm to vacuoles by generating the electrochemical gradient force. Generally, under salinity stress, salt-tolerant plants maintain higher K^+^/Na^+^ or lower Na^+^/K^+^ ratios in the cytoplasm and regulate the osmotic balance of the cells by sequestering Na^+^ in the vacuoles (Maeshima, 2000). Our results are consistent with those of a previous study that a lower Na^+^/K^+^ ratio was observed in tolerant genotypes (Table 1) (Wu et al., 2011; Zahra et al., 2014). The *HvNHX2* and *HvNHX4* isoforms were also induced by salt stress, although not to the same extent as *HvNHX1* and *HvNHX3*. Expression of *HvNHX2* and *HvNHX4* in roots under moderate salt stress was more upregulated in XZ16 than in CM72, whereas high expression levels of *HvNHX2* and *HvNHX4* under severe salt stress in both roots and leaves were observed in CM72. The results from previous studies showed that *HvNHX2* and *HvNHX4* play an important role in the maintenance of K^+^ concentration in plant tissues (Venema et al., 2003; Rodriguez Rosales et al., 2008).

To study the polymorphism in terms of SNP detection in the genes of interest is a powerful technique to investigate the gene(s) function and get desirable mutations for crop breeding. In our previous studies, more allelic variation in Tibetan wild barley accessions was observed for *HvCBF3*, *HvCBF4*, and *HvHKT* genes responsible for salt tolerance (Qui et al., 2011; Wu et al., 2011). These findings are further confirmed by the present study that Tibetan wild barley could provide rich source of allelic variation for the salt-responsive gene(s) as compared with salt-tolerant cultivated barley CM72. So, evaluation of genetic variation and identification of salt tolerance mechanism in wild barley are important steps to unravel the novel alleles involved in salinity tolerance. In conclusion, physiological and gene expression analysis revealed that *HvNHX1* and *HvNHX3* are the candidate genes that function as regulating ions by sequestration of Na^+^ in the vacuole. Moreover, Tibetan wild barley could be used as a rich source of genetic variation to explore the dynamics of abiotic stress tolerance in barley and other cereal crops.

## Data Availability

The authors acknowledge that the data presented in this study must be deposited and made publicly available in an acceptable repository, prior to publication. Frontiers cannot accept a article that does not adhere to our open data policies.

## References

[B1] Apse M. P. Blumwald E. (2007). Na+ Transport in Plants. FEBS Lett. 581, 2247–2254. 10.1016/j.febslet.2007.04.014 17459382

[B2] Barragán V. Leidi E. O. Andrés Z. Rubio L. De Luca A. Fernández J. A. (2012). Ion Exchangers NHX1 and NHX2 Mediate Active Potassium Uptake into Vacuoles to Regulate Cell Turgor and Stomatal Function in Arabidopsis. Plant Cell 24, 1127–1142. 10.1105/tpc.111.095273 22438021 PMC3336136

[B3] Bassil E. Coku A. Blumwald E. (2012). Cellular Ion Homeostasis: Emerging Roles of Intracellular NHX Na+/H+ Antiporters in Plant Growth and Development. J. Exp. Bot. 63, 5727–5740. 10.1093/jxb/ers250 22991159

[B4] Basu S. Kumar A. Benazir I. Kumar G. (2020). Reassessing the Role of Ion Homeostasis for Improving Salinity Tolerance in Crop Plants. Physiologia Plantarum 171, 502–519. 10.1111/ppl.13112 32320060

[B5] Brett C. L. Donowitz M. Rao R. (20052004). Evolutionary Origins of Eukaryotic Sodium/proton Exchangers. Am. J. Physiol. Cel Physiol. 288, C223–C239. 10.1152/ajpcell.0036010.1152/ajpcell.00360.2004 15643048

[B6] Brini F. Hanin M. Mezghani I. Berkowitz G. A. Masmoudi K. (2007). Overexpression of Wheat Na^+^/H^+^ Antiporter *NHX1* and H- Pyrophosphatase *TVP1* Improve Salt- and Drought-Stress Tolerance in *Arabidopsis thaliana* Plants. J. Exp. Bot. 58, 301–308. 17229760 10.1093/jxb/erl251

[B7] Dai F. Nevo E. Wu D. Comadran J. Zhou M. Qiu L. (2012). Tibet Is One of the Centers of Domestication of Cultivated Barley. Proc. Natl. Acad. Sci. 109, 16969–16973. 10.1073/pnas.1215265109 23033493 PMC3479512

[B8] Ellis R. P. Forster B. P. Robinson D. Handley L. L. Gordon D. C. Russell J. R. (2000). Wild Barley: a Source of Genes for Crop Improvement in the 21st century. J. Exp. Bot. 51342, 9–17. 10.1093/jexbot/51.342.9 10938791

[B9] Feuillet C. Langridge P. Waugh R. (2008). Cereal Breeding Takes a Walk on the Wild Side. Trends Genet. 24, 24–32. 10.1016/j.tig.2007.11.001 18054117

[B10] Flowers T. J. Colmer T. D. (2008). Salinity Tolerance in Halophytes*. New Phytol. 179, 945–963. 10.1111/j.1469-8137.2008.02531.x 18565144

[B11] Flowers T. J. (2004). Improving Crop Salt Tolerance. J. Exp. Bot. 55, 307–319. 10.1093/jxb/erh003 14718494

[B12] Fukuda A. Nakamura A. Tagiri A. Tanaka H. Miyao A. Hirochika H. (2004). Function, Intracellular Localization and the Importance in Salt Tolerance of a Vacuolar Na+/H+ Antiporter from Rice. Plant Cel Physiol 45, 146–159. 10.1093/pcp/pch014 14988485

[B13] Gálvez F. J. Baghour M. Hao G. Cagnac O. Rodríguez-Rosales M. P. Venema K. (2012). Expression of *LeNHX* Isoforms in Response to Salt Stress in Salt Sensitive and Salt Tolerant Tomato Species. Plant Physiol. Biochem. 51, 109–115. 10.1016/j.plaphy.2011.10.012 22153246

[B14] He C. Yan J. Shen G. Fu L. Holaday A. S. Auld D. (2005). Expression of an Arabidopsis Vacuolar Sodium/Proton Antiporter Gene in Cotton Improves Photosynthetic Performance under Salt Conditions and Increases Fiber Yield in the Fieldfiber Yield in the Field. Plant Cel Physiol 46, 1848–1854. 10.1093/pcp/pci201 16179357

[B47] Jabeen Z. Fayyaz H. A. Irshad F. Hussain N. Hassan M. N. Li J. (2021). Sodium Nitroprusside Application Improves Morphological and Physiological Attributes of Soybean (Glycine max L.) Under Salinity Stress. PLoS ONE 16 (4), e0248207. 10.1371/journal.pone.0248207 33861749 PMC8051766

[B15] Liu H. Tang R. Zhang Y. Wang C. Lv Q. Gao X. (2010). AtNHX3 Is a Vacuolar K+/H+ Antiporter Required for Low-Potassium Tolerance in *Arabidopsis thaliana* . Plant Cel Environ 33, 1989–1999. 10.1111/j.1365-3040.2010.02200.x 20573049

[B16] Livak K. J. Schmittgen T. D. (2001). Analysis of Relative Gene Expression Data Using Real-Time Quantitative PCR and the 2−ΔΔCT Method. Methods 25 (4), 402–408. 10.1006/meth.2001.1262 11846609

[B17] Maeshima M. (2000). Vacuolar H+-pyrophosphatase. Biochim. Biophys. Acta (Bba) - Biomembranes 1465, 37–51. 10.1016/s0005-2736(00)00130-9 10748246

[B18] Mane A. V. Karadge B. A. Samant J. S. (2010). Salinity Induced Changes in Photosynthetic Pigments and Polyphenols of *Cymbopogon Nardus* (L.) Rendle. J. Chem. Pharm. Res. 2, 338–347.

[B19] Mishra A. Tanna B. (2017). Halophytes: Potential Resources for Salt Stress Tolerance Genes and Promoters. Front. Plant Sci. 8, 829. 10.3389/fpls.2017.00829 28572812 PMC5435751

[B20] Munns R. James R. A. Läuchli A. (2006). Approaches to Increasing the Salt Tolerance of Wheat and Other Cereals. J. Exp. Bot. 57, 1025–1043. 10.1093/jxb/erj100 16510517

[B21] Munns R. Tester M. (2008). Mechanisms of Salinity Tolerance. Annu. Rev. Plant Biol. 59, 651–681. 10.1146/annurev.arplant.59.032607.092911 18444910

[B22] Nevo E. Apelbaum-Elkaher I. Garty J. Beiles A. (1997). Natural Selection Causes Microscale Allozyme Diversity in Wild Barley and a Lichen at 'Evolution Canyon', Mt. Carmel, Israel. Heredity 78, 373–382. 10.1038/hdy.1997.60

[B23] Nevo E. Chen G. (2010). Drought and Salt Tolerances in Wild Relatives for Wheat and Barley Improvement. Plant Cel. Envir. 33, 670–685. 10.1111/j.1365-3040.2009.02107.x 20040064

[B24] Pardo J. M. Cubero B. Leidi E. O. Quintero F. J. (2006). Alkali Cation Exchangers: Roles in Cellular Homeostasis and Stress Tolerance. J. Exp. Bot. 57, 1181–1199. 10.1093/jxb/erj114 16513813

[B25] Paroutis P. Touret N. Grinstein S. (2004). The pH of the Secretory Pathway: Measurement, Determinants, and Regulation. Physiology 19, 207–215. 10.1152/physiol.00005.2004 15304635

[B26] Qiu L. Wu D. Ali S. Cai S. Dai F. Jin X. (2011). Evaluation of Salinity Tolerance and Analysis of Allelic Function of *HvHKT1* and *HvHKT2* in Tibetan Wild Barley. Theor. Appl. Genet. 122, 695–703. 10.1007/s00122-010-1479-2 20981400

[B27] Rodriguez-Rosales M. P. Jiang X. Galvez F. J. Aranda M. N. Cubero B. Venema K. (2008). Overexpression of the Tomato K^+^/H^+^ Antiporter *LeNHX2* Confers Salt Tolerance by Improving Potassium Compartmentalization. New Phytol. 179, 366e377. 10.1111/j.1469-8137.2008.02461.x 19086176

[B28] Rostoks N. Mudie S. Cardle L. Russell J. Ramsay L. Booth A. (2005). Genome-wide SNP Discovery and Linkage Analysis in Barley Based on Genes Responsive to Abiotic Stress. Mol. Genet. Genomics. 274, 515–527. 10.1007/s00438-005-0046-z 16244872

[B29] Russell J. Booth A. Fuller J. Harrower B. Hedley P. Machray G. (2004). A Comparison of Sequence-Based Polymorphism and Haplotype Content in Transcribed and Anonymous Regions of the Barley Genome. Genome 47, 389–398. 10.1139/g03-125 15060592

[B30] Saqib M. Zörb C. Rengel Z. Schubert S. (2005). The Expression of the Endogenous Vacuolar Na+/H+ Antiporters in Roots and Shoots Correlates Positively with the Salt Resistance of Wheat (*Triticum aestivum* L.). Plant Sci. 169, 959–965. 10.1016/j.plantsci.2005.07.001

[B31] Shavrukov Y. Gupta N. K. Miyazaki J. Baho M. N. Chalmers K. J. Tester M. (2010). HvNax3-a Locus Controlling Shoot Sodium Exclusion Derived from Wild Barley (Hordeum Vulgare Ssp. Spontaneum). Funct. Integr. Genomics 10, 277–291. 10.1007/s10142-009-0153-8 20076983

[B32] Shavrukov Y. (2013). Salt Stress or Salt Shock: Which Genes Are We Studying. Exbotj 64, 119–127. 10.1093/jxb/ers316 23186621

[B33] Stepien P. Klobus G. (2005). Antioxidant Defense in the Leaves of C3 and C4 Plants under Salinity Stress. Physiol. Plant 125, 31–40. 10.1111/j.1399-3054.2005.00534.x

[B34] Steppuhn H. M. Genuchten T. Grieve C. M. (2005). Root-zone Salinity Selecting a Product-Yield index and Response Functions for Crop Tolerance. Crop Sci. 45, 209–220. 10.2135/cropsci2005.0209

[B35] Tester M. Davenport R. (2003). Na+ Tolerance and Na+ Transport in Higher Plants. Ann. Bot. 91, 503–527. 10.1093/aob/mcg058 12646496 PMC4242248

[B36] Uçarlı C. Gürel F. (2020). Differential Physiological and Molecular Responses of Three-Leaf Stage Barley (*Hordeum Vulgare* L.) under Salt Stress within Hours. Plant Biotechnol. Rep. 14, 89

[B37] Venema K. Belver A. Marin-Manzano M. C. Rodriguez-Rosales M. P. Donaire J. P. (2003). A Novel Intracellular K^+^/H^+^ Antiporter Related to Na^+^/H^+^ Antiporters Is Important for K Ion Homeostasis in Plants. J. Biol. Chem. 278, 22453e22459. 10.1074/jbc.m210794200 12695519

[B38] Vera-Estrella R. Barkla B. J. García-Ramírez L. Pantoja O. (2005). Salt Stress in Thellungiella Halophila Activates Na+ Transport Mechanisms Required for Salinity Tolerance. Plant Physiol. 139, 1507–1517. 10.1104/pp.105.067850 16244148 PMC1283785

[B39] Wani S. H. Kumar V. Khare T. Guddimalli R. Parveda M. Solymosi K. (2020). Engineering Salinity Tolerance in Plants: Progress and Prospects. Planta 251, 76–29. 10.1007/s00425-020-03366-6 32152761

[B40] Wu D. Qiu L. Xu L. Ye L. ChenSun M. D. Sun D. (2011). Genetic Variation of *HvCBF* Genes and Their Association with Salinity Tolerance in Tibetan Annual Wild Barley. PLoS ONE 6, e22938. 10.1371/journal.pone.0022938 21829562 PMC3145780

[B41] Yamaguchi T. Blumwald E. (2005). Developing Salt-Tolerant Crop Plants: Challenges and Opportunities. Trends Plant Sci. 10, 615–620. 10.1016/j.tplants.2005.10.002 16280254

[B43] Zahra J. Nazim H. Cai S. Han Y. Wu D. Zhang B. (2014). The Influence of Salinity on Cell Ultrastructures and Photosynthetic Apparatus of Barley Genotypes Differing in Salt Stress Tolerance. Acta Physiol. Plant 36, 1261–1269. 10.1007/s11738-014-1506-z

[B44] Zhang H.-X. Blumwald E. (2001). Transgenic Salt-Tolerant Tomato Plants Accumulate Salt in Foliage but Not in Fruit. Nat. Biotechnol. 19, 765–768. 10.1038/90824 11479571

[B45] Zhu J.-K. (2001). Plant Salt Tolerance. Trends Plant Sci. 6, 66–71. 10.1016/s1360-1385(00)01838-0 11173290

[B46] Zhu J. K. (2007). “Plant Salt Stress,” in Encyclopedia of Life Sciences. Editor Wiley J.. 10.1002/9780470015902.a0001300.pub2

