# Supplementary material for: NHX-Type Na+/H+ Antiporter Gene Expression Under Different Salt Levels and Allelic Diversity of HvNHX in Wild and Cultivated Barleys
Source: Front Genet. 2022 Feb 22;12:809988. doi: 10.3389/fgene.2021.809988 (PMC8902669; doi:10.3389/fgene.2021.809988)
Supplement: Supplementary file 1 [file Table1.DOCX]

Supplementary Material

Supplementary Table 1 primers used for RT-PCR analysis

| **Gene** | **Amplicon size (bp)** | **Forward primer 5′-3′** | **Reverse primer 5′-3′** |
| --- | --- | --- | --- |
| *HvNHX1* | 274 | TCCAGGTGAAGAAGAAGCAG | GTGGCATCGTTCACAACAC |
| *HvNHX2* | 227 | GGCTATCTTCTCAGCAACC | CAAGAACGGTGCTGGTGAG |
| *HvNHX3* | 315 | CGATGCGACATCAGTTGTG | CAGGTATAGTGCGACATTAC |
| *HvNHX4* | 344 | AGGAGTAATGCAGCAGGAG | GTTTCAGCCAAAGATGATAGC |

Supplementary Table 2 primers used for SNP analysis

| **Gene** | **Amplicon size (bp)** | **Forward primer 5′-3′** | **Reverse primer 5′-3′** |
| --- | --- | --- | --- |
| *HvNHX1* | P1: 753 | CGTCGATTCGTCTCCGGATT | ACGACTGATGTGGCATCGTT |
|  | P2: 308 | TCACAATCTCTCTTGCTGCC | AAGGAAGGTGCTTGACACGA |
|  | P3: 658 | TGCTCATGGCCTACCTCTCA | TCCATGTCCGAGCCTAGCA |
|  | P4: 626 | TTTGCTAGTGACAGCCCTGG | CTCCACAACGTTCATGCCAC |
| *HvNHX3* | P1: 744 | GGAATAATGGGGTTGGGGCT | TTCACGATCAGTGGAGTGCC |
|  | P2: 947 | ACATCGCACTTGGGGCAATA | GTGCCTCGCTGGAATCAGTA |
|  | P3: 520 | TTATTTGGTGGGCGGGTCTC | TAAACCCTCCCCTTCTGCGT |

Supplementary Figure1 Positions of primers on the *HvNHX1* gene

Supplementary Figure1 Positions of primers on the *HvNHX3* gene
